# Supplementary material for: Resistance to post-emergent herbicides is becoming common for grass weeds on New Zealand wheat and barley farms
Source: PLoS One. 2021 Oct 14;16(10):e0258685. doi: 10.1371/journal.pone.0258685 (PMC8516262; doi:10.1371/journal.pone.0258685)
Supplement: Attachment — Submitted filename: PONE-D-21-22929_revised.pdf [file pone.0258685.s002.pdf]

PLOS ONE

Resistance to post-emergent herbicides is becoming common for grass weeds on New Zealand wheat and barley farms.

--Manuscript Draft--

**Manuscript Number:**	PONE-D-21-22929
**Article Type:**	Research Article
**Full Title:**	Resistance to post-emergent herbicides is becoming common for grass weeds on New Zealand wheat and barley farms.
**Short Title:**	Herbicide resistance on New Zealand wheat and barley farms.
**Corresponding Author:**	Christopher E. Buddenhagen, Ph.D<br>AgResearch Ltd Ruakura Research Centre<br>Hamilton, NEW ZEALAND
**Keywords:**	clodinafop; fenoxaprop; haloxyfop; pinoxaden; clethodim; iodosulfuron; pyroxsulam; glyphosate; Acetyl CoA Carboxylase (ACCase); Acetolactate Synthase (ALS); Acetohydroxy Acid Synthase (AHAS)
**Abstract:**	To estimate the prevalence of herbicide-resistant weeds, we randomly surveyed 87 wheat and barley farms in the Canterbury region of New Zealand. We collected over 600 weed seed samples from 1-10 mother plants per taxon visible to searchers immediately prior to harvest (two fields per farm). We also tested some samples provided by agronomists on an ad-hoc basis. Over 40,000 seedlings were grown to the 2-4 leaf stage in glasshouse conditions and sprayed with high priority herbicides for grasses from the three modes-of-action (ACCase-inhibitors, ALS-inhibitors, and glyphosate). We applied the highest manufacturer recommended label rates for the products registered for use in New Zealand, often higher than the discriminatory rates used in studies elsewhere. Published studies of resistance were rare in New Zealand but we found weeds survived herbicide applications on 42 of the 87 (48%) farms, while susceptible reference populations died. We report percent of resistant farms (denominator is 87 farms) for ACCase-inhibitors, ALS-inhibitors, and glyphosate respectively as: *Avena fatua* (9%, 1%, 0% of farms), *Bromus catharticus* (0%, 2%, 0%), *Lolium* spp. (17%, 28%, 0%), *Phalaris minor* (1%, 6%, 0%), and *Vulpia bromoides* (0%, not tested, 0%). Not all farms had the weeds present, five had no obvious weeds prior to harvest. This survey revealed New Zealand's first documented cases of resistance in *Phalaris minor* (fenoxaprop, clodinafop, iodosulfuron) and *Bromus catharticus* (pyroxsulam). Twelve of the 87 randomly sampled farms (14%) had ALS-inhibitor chlorsulfuron-resistant sow thistles, mostly *Sonchus asper* but also *Sonchus oleraceus*. We confirmed resistance from industry-supplied samples of the grasses *Digitaria sanguinalis* (nicosulfuron, two farms), *Phalaris minor* (iodosulfuron, one farm), and *Lolium* spp. (cases included glyphosate, haloxyfop, pinoxaden, iodosulfuron, and pyroxsulam, 9 farms). Industry also supplied *Stellaria media* samples which were confirmed resistant to chlorsulfuron and flumetsulam (ALS inhibitors) sourced from clover and ryegrass fields from the North and South Island.
**Order of Authors:**	Christopher E. Buddenhagen, Ph.D
	Trevor K. James
	Zachary Ngow
	Deborah L. Hackell
	M. Phil Rolston
	Richard J Chynoweth
	Matilda Gunnarsson
	Fengshuo Li
	Kerry C. Harrington
	Hossein Ghanizadeh

Powered by Editorial Manager® and ProduXion Manager® from Aries Systems Corporation

Additional Information:	
**Question**	**Response**
**Financial Disclosure**<br><br>Enter a financial disclosure statement that describes the sources of funding for the work included in this submission. Review the submission guidelines for detailed requirements. View published research articles from *PLOS ONE* for specific examples.<br><br>This statement is required for submission and **will appear in the published article** if the submission is accepted. Please make sure it is accurate.<br><br>**Unfunded studies**<br>Enter: *The author(s) received no specific funding for this work.*<br><br>**Funded studies**<br>Enter a statement with the following details:<br>• Initials of the authors who received each award<br>• Grant numbers awarded to each author<br>• The full name of each funder<br>• URL of each funder website<br>• Did the sponsors or funders play any role in the study design, data collection and analysis, decision to publish, or preparation of the manuscript?<br>• **NO** - Include this sentence at the end of your statement: *The funders had no role in study design, data collection and analysis, decision to publish, or preparation of the manuscript.*<br>• **YES** - Specify the role(s) played.<br><br>* typeset	All the authors worked under the Ministry of Business, Innovation and Employment [grant number C10X1806] to AgResearch Ltd.: “Improved weed control and vegetation management to minimize future herbicide resistance.” AgResearch Ltd is a crown (i.e., the Government) owned research institution in New Zealand.<br><br>https://www.mbie.govt.nz/assets/e5a12d67b2/2018-endeavour-round-successful-projects.pdf<br><br>The funders had no role in study design, data collection and analysis, decision to publish, or preparation of the manuscript.
**Competing Interests**<br><br>Use the instructions below to enter a competing interest statement for this submission. On behalf of all authors, disclose any competing interests that could be perceived to bias this work—acknowledging all financial support and any other relevant financial or non-financial competing interests.	The authors have declared that no competing interests exist.

This statement is required for submission and **will appear in the published article** if the submission is accepted. Please make sure it is accurate and that any funding sources listed in your Funding Information later in the submission form are also declared in your Financial Disclosure statement.<br><br>View published research articles from *PLOS ONE* for specific examples.<br><br>**NO authors have competing interests**<br><br>Enter: *The authors have declared that no competing interests exist.*<br><br>**Authors with competing interests**<br><br>Enter competing interest details beginning with this statement:<br><br>*I have read the journal's policy and the authors of this manuscript have the following competing interests: [insert competing interests here]*<br><br>* typeset	
**Ethics Statement**<br><br>Enter an ethics statement for this submission. This statement is required if the study involved:<br><br>• Human participants<br>• Human specimens or tissue<br>• Vertebrate animals or cephalopods<br>• Vertebrate embryos or tissues<br>• Field research<br><br>Write "N/A" if the submission does not require an ethics statement.<br><br>General guidance is provided below. Consult the submission guidelines for detailed instructions. **Make sure that all information entered here is included in the**	The Foundation for Arable Research obtained permission from the member farmers to collect weed seeds and plants.

Powered by Editorial Manager® and ProduXion Manager® from Aries Systems Corporation

<table>
<tr><td>

Methods section of the manuscript.

Format for specific study types

Human Subject Research (involving human participants and/or tissue)

- Give the name of the institutional review board or ethics committee that approved the study
- Include the approval number and/or a statement indicating approval of this research
- Indicate the form of consent obtained (written/oral) or the reason that consent was not obtained (e.g. the data were analyzed anonymously)

Animal Research (involving vertebrate animals, embryos or tissues)

- Provide the name of the Institutional Animal Care and Use Committee (IACUC) or other relevant ethics board that reviewed the study protocol, and indicate whether they approved this research or granted a formal waiver of ethical approval
- Include an approval number if one was obtained
- If the study involved *non-human primates*, add *additional details* about animal welfare and steps taken to ameliorate suffering
- If anesthesia, euthanasia, or any kind of animal sacrifice is part of the study, include briefly which substances and/or methods were applied

Field Research

Include the following details if this study involves the collection of plant, animal, or other materials from a natural setting:

- Field permit number
- Name of the institution or relevant body that granted permission

</td><td></td></tr>
<tr><td>

Data Availability

Authors are required to make all data underlying the findings described fully available, without restriction, and from the time of publication. PLOS allows rare exceptions to address legal and ethical concerns. See the PLOS Data Policy and FAQ for detailed information.

</td><td>No - some restrictions will apply</td></tr>
</table>

A Data Availability Statement describing where the data can be found is required at submission. Your answers to this question constitute the Data Availability Statement and **will be published in the article**, if accepted.<br><br>**Important:** Stating 'data available on request from the author' is not sufficient. If your data are only available upon request, select 'No' for the first question and explain your exceptional situation in the text box.<br><br>Do the authors confirm that all data underlying the findings described in their manuscript are fully available without restriction?	
**Describe where the data may be found in full sentences. If you are copying our sample text, replace any instances of XXX with the appropriate details.**<br><br>• If the data are **held or will be held in a public repository**, include URLs, accession numbers or DOIs. If this information will only be available after acceptance, indicate this by ticking the box below. For example: *All XXX files are available from the XXX database (accession number(s) XXX, XXX.*).<br>• If the data are all contained **within the manuscript and/or Supporting Information files**, enter the following: *All relevant data are within the manuscript and its Supporting Information files.*<br>• If neither of these applies but you are able to provide **details of access elsewhere**, with or without limitations, please do so. For example:<br><br>*Data cannot be shared publicly because of [XXX]. Data are available from the XXX Institutional Data Access / Ethics Committee (contact via XXX) for researchers who meet the criteria for access to confidential data.*<br><br>*The data underlying the results*	The data are provided about herbicide trials, but the exact location information for farms with or without herbicide resistance is not. If someone legitimately wants to repeat the survey work, we would provide it.

presented in the study are available from (include the name of the third party and contact information or URL).<br>• This text is appropriate if the data are owned by a third party and authors do not have permission to share the data.<br><br>* typeset	
Additional data availability information:	

Manuscript

Click here to access/download;Manuscript;Buddenhagen et al 2021 HR Submitted.docx

Resistance to post-emergent herbicides is becoming common for grass weeds on New Zealand wheat and barley farms.

Christopher E. Buddenhagen[1], **Trevor K. James**[1], **Zachary Ngow**[1], **Deborah L. Hackell**[1], **M. Phil Rolston**[2], **Richard J. Chynoweth**[2], **Matilda Gunnarsson**[2], **Fengshuo Li**[3,4], **Kerry C. Harrington**[4], **Hossein Ghanizadeh**[4]

[1] AgResearch Ltd., Hamilton, New Zealand

[2] Foundation for Arable Research, Christchurch, New Zealand

[3] College of Horticulture and Landscape Architecture, Northeast Agricultural University, Harbin 150030, China

[4] School of Agriculture and Environment, Massey University, Palmerston North, New Zealand

Correspondence

Christopher E. Buddenhagen, AgResearch Ltd., Hamilton, New Zealand,

Email: Chris.Buddenhagen@agresearch.co.nz

Hossein Ghanizadeh, Massey University, Palmerston North, New Zealand

Email: h.ghanizadeh@massey.ac.nz

Abstract

To estimate the prevalence of herbicide-resistant weeds, we randomly surveyed 87 wheat and barley farms in the Canterbury region of New Zealand. We collected over 600 weed seed samples from 1-10 mother plants per taxon visible to searchers immediately prior to harvest (two fields per farm). We also tested some samples provided by agronomists on an ad-hoc basis. Over 40,000 seedlings were grown to the 2-4 leaf stage in glasshouse conditions and sprayed with high priority herbicides for grasses from the three modes-of-action (ACCase-inhibitors, ALS-inhibitors, and glyphosate). We applied the highest manufacturer recommended label rates for the products registered for use in New Zealand, often higher than the discriminatory rates used in studies elsewhere. Published studies of resistance were rare in New Zealand but we found weeds survived herbicide applications on 42 of the 87 (48%) farms, while susceptible reference populations died. We report percent of resistant farms (denominator is 87 farms) for ACCase-inhibitors, ALS-inhibitors, and glyphosate respectively as: *Avena fatua* (9%, 1%, 0% of farms), *Bromus catharticus* (0%, 2%, 0%), *Lolium* spp. (17%, 28%, 0%), *Phalaris minor* (1%, 6%, 0%), and *Vulpi*[illegible]*omoides* (0%, not tested, 0%). Not all farms had the weeds present, five had no obvious weeds prior to harvest. This survey revealed New Zealand's first documented cases of resistance in *Phalaris minor* (fenoxaprop, clodinafop, iodosulfuron) and *Bromus catharticus* (pyroxsulam). Twelve of the 87 randomly sampled farms (14%) had ALS-inhibitor chlorsulfuron-resistant sow thistles, mostly *Sonchus asper* but also *Sonchus oleraceus*. We confirmed resistance from industry-supplied samples of the grasses *Digitaria sanguinalis* (nicosulfuron, two farms), *Phalaris minor* (iodosulfuron, one farm), and *Lolium* spp. (cases included glyphosate, haloxyfop, pinoxaden, iodosulfuron, and pyroxsulam, 9 farms). Industry also supplied *Stellaria media* samples which were confirmed resistant to chlorsulfuron and flumetsulam (ALS inhibitors) sourced from clover and ryegrass fields from the North and South Island.

KEYWORDS

clodinafop, fenoxaprop, haloxyfop, pinoxaden, clethodim, iodosulfuron, pyroxsulam, glyphosate

Introduction

Weed control programs that use herbicides have proven to be cost-effective for improving yields of staple crops by an average of 30% [1] and typically provide a 2-4-fold economic return [2]. They are also a key element in no-till planting programs for wheat and barley farms in New Zealand that improve soil structure and prevent soil loss through erosion [3]. Nevertheless, farmer practices worldwide have led to the selection of weeds with infrequent genetic mutations that confer resistance to the herbicides, allowing weeds to escape control, reproduce and form resistant populations [4]. Globally, resistance is common in wheat (344 cases and 83 species) and barley (87 cases, 47 species) crops and in arable crops generally [5]. Based on worldwide patterns of resistance, Ngow et al [6] identified 16 species with a high risk of developing resistance in New Zealand wheat and barley fields (8 were grasses *Avena fatua* L., *Avena sterilis* L., *Digitaria sanguinalis* (L.) Scop.,*Echinochloa crus-galli* (L.) P. Beauv. , *Lolium multiflorum* Lam., *Lolium perenne* L., *Phalaris minor* Retz., and *Poa annua* L.; these grasses were in the top 10 for risk of developing herbicide resistance).

In any given year >50% of New Zealand arable production areas are under wheat (~45000 ha) and barley (~55000 ha) rotations [7]–and only a small proportion of the more than 800 farms are registered as organic [<50 certified farms; ,8]. Under intense management, production levels are high, with farmers in New Zealand obtaining world record yields of wheat (17.39 tons/ha) and barley (13.8 tons/ha) in 2017 and 2015, respectively [9,10]. Yet only a few instances of herbicide resistance in ryegrass species *Lolium perenne* and *Lolium multiflorum* have been documented to date in wheat and barley in New Zealand [11] and in *Avena fatua* [12]. There is at least one case of *Stellaria media* (L.) Vill. resistance recorded in an oat crop [13]. Compared to Australia or the United States, there appears to be only a small number of resistance cases documented in New Zealand arable farms [14]. Frequent crop rotations [15] may allow New Zealand farmers to rotate herbicidal modes-of-action (e.g., effective against broadleaf or grass weeds), implement resting periods, stale seed bed or cultivation steps; these are widely regarded as key elements in best practice for resistance management [16]. Species commonly included in wheat and barley rotations in New Zealand are pasture, spring-sown peas, linseed, ryegrass, clover, oilseed rape, and wheat or barley. The higher than usual manufacturer label recommended application rates in New Zealand (compared to Australia or USA) could also have an influence on the rates of resistance development (discussed later). Another plausible explanation for the low number of

resistance cases in wheat and barley farms in New Zealand is that the problem is simply under-investigated.

We set out to determine the prevalence of herbicide-resistant weeds on 87 arable farms with wheat and barley rotations in a northern (near Lincoln) and southern locality (near Timaru) of the Canterbury region in the South Island of New Zealand. Farms were randomly selected, and sample collections focused on weeds that survived weed control measures and had mature seeds just before wheat and barley harvest. As grass weeds were the most common, we focus our reporting on those (but mention other cases). This work represents the first random survey to detect herbicide resistance for any agricultural sector in New Zealand.

Materials and Methods

Collection of plant material

Weeds seeds were collected from 87 randomly selected arable farms from the Foundation for Arable Research (FAR) member database in January and February 2019 and 2020. This represents 21% of the possible farms in the selected regions. In 2019, we surveyed 52 farms between the Rakaia and Waimakariri Rivers near Lincoln, and in 2020, we visited 35 farms near Timaru. The FAR member database is thought to contain at least 90% of arable farmers in New Zealand. Seeds were collected from one or two fields per farm, usually with wheat or barley, or more rarely clover seed crops. If weeds were present, seed from 1-10 individual weeds with viable seed were collected for each weed species (grass weeds were the most common and the focus of this study). If weeds for a species were frequent in a field, an effort was made to space out the collections from across the whole field. Plants growing in mid-field (as opposed to edges) were favoured. In 2019, seed from each species was collected and bulked together for a field sample. *Lolium multiflorum* and *Lolium perenne* seed was separated based on field determinations of species (based on awn length and leaf blade width). However, most ryegrass seed samples were found to be difficult to distinguish, and many were hybrids. In 2020, 35 farms, primarily from Southern Canterbury centred around Timaru, were surveyed. Unlike 2019, in 2020 we kept separate seed samples for each mother plant. Seed samples were labelled with location and species information and stored in paper envelopes or bags and kept in a cool store at 4°C until planting. A single georeferenced point was recorded for each field sampled. For this paper, we focus our results on the grasses and comment on our results for a few other cases, including some detected via ad-hoc industry supplied samples.

After advising that our herbicide resistance project provided free testing, industry representatives and agronomists sent us seeds from several suspected resistant plants i.e., that were not part of the random survey. We tested ryegrass from additional 11 farms with suspected resistance to a variety of herbicides, including pyroxsulam, pinoxaden and one case of glyphosate resistance (mostly from wheat and barley fields). We were also supplied with two samples of *Stellaria media* suspected of resistance to flumetsulam (ALS inhibitor). One sample from barley in the South Island and one from ryegrass seed crops in the North Island. Additionally, *Digitaria sanguinalis* with suspected resistance to nicosulfuron (ALS inhibitor) from a maize crop from the Waikato region of the North Island. *Avena fatua* from three farms in Canterbury were suspected of pinoxaden and pyroxsulam resistance (ACCase inhibitor and ALS inhibitor respectively). A sample of *Phalaris minor* suspected of being resistant to ALS-inhibitors was also provided for testing (also from Canterbury.

Growing plants and spraying

Plants grown at Ruakura

At the Ruakura Research Centre, Hamilton, we planted 10-40 (usually 30) weed seeds per pot in March 2019 from each sample in three to six 9 cm x 9 cm x 9 cm black plastic pots (one per herbicide that we tested) containing sterile, commercial potting mix (Daltons) that included a slow-release fertilizer. A susceptible control was also grown. Pots were kept moist (watered every 2-3 days) and kept in a temperature regulated glasshouse at Ruakura and maintained at between 18 and 25°C. In April and May 2020, we changed our protocol. 20 seeds were sown into propagation trays (22 cm x 35 cm x 5 cm) into one of six rows or lanes, such that each tray could contain four to five field collected samples and a known herbicide susceptible control and a known herbicide resistant control, if available. Again, each sample would have its seed spread between three to six propagation trays, with one tray per herbicide tested. Samples in these trayswere kept moist (watered every 2-3 days) and kept in a temperature regulated glasshouse at Ruakura and maintained at between 18 and 25°C. Propagation trays with *Lolium* spp. seed were planted at 2-3 mm depth, watered and chilled in a cool store at 4 °C for 48 hours before being placed in the glasshouse. *Avena fatua* samples from 2020 were dehusked and soaked in 0.1% KNO_3 for 24 hours before planting into trays at 3-4 mm depth [e.g., 17]. Grass seedlings were raised to the 2-4 leaf stage before herbicide was applied. Susceptible controls for *Avena fatua, Bromus diandrus* Roth and *Bromus catharticus* Vahl were sourced from an organic farm near Methven. Susceptible *Lolium* spp. samples used in this study is the same as

that described in an earlier New Zealand herbicide resistance study, diploid varieties Trojan and Tabu for *L. perenne* and *L. multiflorum*, respectively [18]. Before herbicide treatment, seedlings in all pots or propagation trays were counted. Known susceptible controls for *Vulpia bromoides* L. Gray and *Phalaris minor* were not available at the time of herbicide treatment, but some samples in every treatment block did show 100% mortality.

Depending on the availability of adequate seed in a sample, the number of herbicides that could be tested changed. Herbicides were tried in the priority order shown for each taxon (Table 1). For example, a sample of 60 seeds would only be tested against the top three to five priority herbicides (Table 1) as we tried to maintain between 10 and 25 seeds per treatment. The same priority order was used for samples treated at Massey University (see below). All herbicide treatments were applied using the highest recommended label rate for the herbicide being tested (Table 1) with a moving belt sprayer using a single TeeJet TT11002 fan nozzle at 200 kPa, positioned 440 mm above the top of the pots/trays to apply 200 litres/ha. We included isoproturon on *Vulpia bromoides* which is a photosystem II inhibitor because industry consultants thought it is effective, even though this species is not mentioned on the herbicide label. In 2019, up to 12 pots were grouped into trays (22cm x 35cm x 5 cm) nursery for spraying. Up to about 22 nursery trays per herbicide treatment could be sprayed with a single herbicide at any given time due to the 1 litre capacity of the spray tank reservoir. Only one or two susceptible controls (in individual pots) were used per herbicide treatment. In 2020, as mentioned above seeds were planted out in lanes across each tray with a susceptible control in one of the lanes per propagation tray. *Sonchus oleraceus* L. and *Sonchus asper* (L.) Hill from the random surveys were treated with chlorsulfuron 20 g ai/ha (AgPro Chloro®) with a non-ionic surfactant (0.1%).

We also report results from a few other weeds supplied to us by industry agronomists that were tested. *Stellaria media* samples were sourced from ryegrass rotation (near Matamata on the North Island) and one sample from Ashburton in the South Island from a field planted in clover and ryegrass were treated with flumetsulam 30 g ai/ha (Preside®) and a paraffinic oil surfactant (0.5%), and chlorsulfuron 20 g ai/ha (AgPro Chloro®) with a non-ionic surfactant (0.25%). *Digitaria sanguinalis* supplied to us from two maize farms near Matamata on the North Island was treated with nicosulfuron 60 g ai/ha with paraffinic oil surfactant (0.5%) (Appendix S1).

Plants grown at Massey University

Avena fatua seed samples from 2019 were processed at Massey University, Palmerston North. To overcome seed dormancy, seed samples were dehusked and soaked in 800 ppm gibberellic acid (GA_3 overnight at room temperature before they were chilled for 3 days at 5 °C. The seeds were then planted into polyethylene planter bags (PB2, 1.2 L) containing potting mix and slow-release fertilizer as described by Ghanizadeh and Harrington [19]. There were three replicates for each population and herbicide combination. Each replicate consisted of 10-17 seeds planted in one pot for most of the populations and herbicide combinations. However, due to limited seeds, for a few populations, there were seven to 10 seeds per replicate. The pots were kept in a glasshouse with a capillary irrigation system. The minimum/maximum daily temperature in the glasshouse was 19.6/22.3 °C and the average relative humidity was 55%. At 4-5 days after planting, 100% emergence was recorded for all replicates. At 10 days after emergence, when the plants were at the 2-leaf stage, they were treated with herbicide. Each herbicide was applied using a dual-nozzle (Teejet 730231 flat-fan nozzles) laboratory track sprayer calibrated to deliver 230 L ha^{-1} of herbicide solution at 200 kPa.

Table 1. Herbicides used and rates of application for the grass weed species mentioned in this article. Herbicides were tried in the priority order shown for each taxon (see methods for detail). Seeds of *Avena* spp. and *Bromus* spp. were replanted and sprayed at the 30 g ai/ha rate for pyroxsulam (Rexade GoDri). Rates used for *Sonchus asper, Sonchus oleraceus, Stellaria media* and *Digitaria sanguinalis* are reported in the text.

Weed	Priority Order	Trade Name	Active Ingredients	Rate g ai per ha	Adjuvant	Adjuvant rate
Lolium perenne	1	Ignite	haloxyfop	250	none	none
Lolium perenne	2	Twinax	pinoxaden	30	Adigor	0.50%
Lolium perenne	3	Rexade	halauxifen-methyl and pyroxsulam	15	Actiwett	0.25%
Lolium perenne	4	Weedmaster	glyphosate	1458	Pulse	0.10%
Lolium perenne	5	Hussar	iodosulfuron	7.5	Partner	0.50%
Lolium perenne	6	Sequence	clethodim	120	Bonza	0.50%
Lolium multiflorum	1	Ignite	haloxyfop	125	none	
Lolium multiflorum	2	Twinax	pinoxaden	30	Adigor	0.50%
	3	Simplicity	pyroxsulam	15	Actiwett	0.25%

Lolium multiflorum	3	Rexade	halauxifen-methyl and pyroxsulam	5/10 and 15/30	Actiwett	0.25%
Lolium multiflorum	4	Weedmaster	glyphosate	1458	Pulse	0.10%
Lolium multiflorum	5	Hussar	iodosulfuron	7.5	Partner	0.50%
Lolium multiflorum	6	Sequence	clethodim	120	Bonza	0.50%
Avena fatua	1	Puma-S	fenoxaprop	51.75	none	
Avena fatua	2	Twinax	pinoxaden	25	Adigor	0.50%
Avena fatua	3	Sequence	clethodim	120	Bonza	0.50%
Avena fatua	4	Simplicity	pyroxsulam	15	Actiwett	0.25%
Avena fatua	4	Rexade	halauxifen-methyl and pyroxsulam	5/10 and 15/30	Actiwett	0.25%
Avena fatua	5	Weedmaster	glyphosate	702	Pulse	0.10%
Bromus catharticus	1	Ignite	haloxyfop	250	none	none
Bromus catharticus	2	Sequence	clethodim	240	Bonza	0.50%
Bromus catharticus	3	Rexade	halauxifen-methyl and pyroxsulam	5/10 and 15/30	Contact	0.25%
Bromus catharticus	4	Weedmaster	glyphosate	540	Pulse	0.10%
Bromus diandrus	1	Ignite	haloxyfop	250	none	
Bromus diandrus	2	Sequence	clethodim	240	Bonza	0.50%
Bromus diandrus	3	Simplicity	pyroxsulam	15	Contact	0.25%
Bromus diandrus	3	Rexade	halauxifen-methyl and pyroxsulam	5/10 and 15/30	Actiwett	0.25%
Bromus diandrus	4	Weedmaster	glyphosate	540	Pulse	0.10%
Bromus hordeaceus	1	Ignite	haloxyfop	250	none	
Bromus hordeaceus	2	Sequence	clethodim	240	Bonza	0.50%
Bromus hordeaceus	3	Simplicity	pyroxsulam	15/30	Contact	0.25%
Bromus hordeaceus	3	Rexade	halauxifen-methyl and pyroxsulam	5/10 and 15/30	Contact	0.25%
Bromus hordeaceus	4	Weedmaster	glyphosate	540	Pulse	0.10%
Phalaris minor	1	Hussar	iodosulfuron	7.5	Partner	0.50%
Phalaris minor	2	Mandate	clodinafop	24	Uptake	0.50%
Phalaris minor	3	Sequence	clethodim	240	Bonza	0.50%
Phalaris minor	4	Weedmaster	glyphosate	702	Pulse	0.10%
Phalaris minor	5	Ignite	haloxyfop	60	none	
Phalaris minor	6	Twinax	pinoxaden	30	Adigor	0.50%

Determining mortality

Mortality was assessed 2 weeks after spraying for the shorter acting herbicides (e.g., glyphosate and haloxyfop) and after 3 weeks for longer acting herbicides (e.g., pyroxsulam, iodosulfuron, clethodim). If plant stem tissue near the base was soft and discoloured or if most of the plant was brown or black, we determined the plant to be dead. In all herbicide resistance cases reported here the susceptible control died if it were available, or some of the samples sprayed at the same time experienced 100% mortality if not, i.e., *Phalaris,* and *Vulpia*.

Statistical analysis

Maps and pivot tables statistics were produced in the R statistical platform using the ggmap and tidyverse packages [20–22]. We were conservative about determining resistance, we excluded samples where germination was poor (<5 seedlings). We determined a farm as surviving treatment (or "resistant") if a sample from it had more than 10% of plants survive and number that survived was greater than three plants. We tested the hypothesis that a higher proportion of the tested Timaru farms (thought to have less crop rotation) had cases of resistance compared to Lincoln using the prop.test function in R [22].

Results

We report on the results from over 600 samples of grasses collected from two sampling regions, including a total of 87 farms that were near mid-northern Canterbury near Lincoln (52 farms) and southern Canterbury near Timaru (35 farms). Each farm would often have more than one crop type, sampled fields included wheat (61 farms), barley (30 farms) and white clover (21), but we included <2 farm fields with linseed, beets, and peas. The following common weedy grasses were found as survivors prior to harvest on a sizeable number of the 87 farms we visited: *Avena fatua* (52 farms), *Bromus diandrus* (16), *Bromus hordeaceus* (19), *Bromus catharticus* (29), *Lolium* spp. for suspected hybrids (23), *Lolium multiflorum* (38), *Lolium perenne* (46), *Lolium* spp. (occurred on a total 57 farms), *Phalaris minor* (17) and *Vulpia bromoides* (14). Only three broadleaf weeds were common, *Sonchus asper* and *S. oleraceus* were found on 27 farms and *Achillea millefolium* L. (yarrow) on five. All other weeds were collected three or fewer times. Some seed samples had poor germination and were not included in our tests of resistance. Primarily we focused on results for the common grasses surviving label rate applications of different post-emergent herbicides but grouped the

results by weed genus and herbicide modes-of-action. More detailed results broken down by farm and herbicide active ingredients are provided in the supplementary materials (Appendix S1). Farms with plants (within each weed genus) surviving treatment with one or more herbicides for a mode-of-action are indicated in Table 2, a spatial presentation of the same data indicates where the farms with resistance were located (Figure 1), and resistance to herbicides in the given mode of action (Figure 2).

Fig 1. Map of the farms in Canterbury, New Zealand, where we tested seedlings of one or more weed species for resistance, with symbols indicating whether the sample was from the random survey, or from ad-hoc reports of resistance. Resistant weeds included *Lolium* spp., *Avena fatua, Phalaris minor, Bromus catharticus, Sonchus asper* and *Sonchus oleraceus*.

Fig 2. Map of where weeds from each genus (vertical panels) survived or died. Treatments involved using one or more herbicides in the herbicide groups indicated (horizontal panels); the specific herbicides used per species in each weed genus are mentioned in the methods and supplementary data.

In the random survey, we detected some form of resistance (for any taxon) on 42 farms (48% of those surveyed), resistance was found for ALS-inhibitors on 35 farms (40%) and to ACCase-inhibitors on 20 (23%) farms. No cases of glyphosate-resistant grasses were detected in the random survey. Only *Vulpia bromoides* was tested for the photosystem II (PSII) inhibiting herbicide isoproturon and no resistance was detected. To strengthen our determination of resistance to a particular herbicide mode of action, multiple herbicides were tested for some modes-of-action depending on the species, ACCase-inhibitors (haloxyfop, clodinafop, fenoxaprop, pinoxaden, clethodim) and two herbicides in the ALS-inhibitors (iodosulfuron and pyroxsulam) were tested. In addition, different weeds were subjected to one or more of these herbicides, in priority order according to the quantity of seed available (Table 1). *Avena fatua* seedlings were often not killed by the application of the ALS-inhibiting herbicide pyroxsulam at the 15 g ai/ha rate but only one farm was confirmed to have resistant plants when sprayed at 30 g ai/ha. While eight (9%) farms had *A. fatua* resistant to ACCase-inhibiting herbicides, samples from six farms survived fenoxaprop but were killed when treated with haloxyfop or clethodim (Appendix S1). In the case of *Lolium* species, we found that it was hard to get an accurate taxonomic identification from the seed samples and doubts about their provenance persisted even though most surviving plants were

grown on to flowering stage. Many ryegrass hybrids are grown in New Zealand, and we attempted to class them based on awn length, mid-rib prominence and lamina width. Nevertheless, we used the higher herbicide rates recommended for *L. perenne* in year 2 of the study (for all *Lolium* plants collected near Timaru). We also documented resistance to ALS- and ACCase-inhibitors for *Phalaris minor*, and to ALS-inhibiting herbicides in *Bromus catharticus* in two adjacent farms. Resistance was found for more than one weed genus in 11 farms, and three of these farms had resistant weeds from three genera. A total of 13 farms showed resistance to ALS- and ACCase-inhibiting herbicides (*A. fatua* for two farms) and (*Lolium* spp. for 12 farms) – two farms had resistance to both ALS- and ACCase-inhibitors for both *Lolium* spp. and *A. fatua* samples. Twelve randomly sampled farms (14%) had chlorsulfuron-resistant sow thistles, mostly *Sonchus asper* but also *Sonchus oleraceus*. We also tested the hypothesis that samples from farms in the Lincoln region were less likely to develop cases of resistance compared with Timaru because they are regarded as having more complex crop rotations. At Lincoln 22 of 52 farms had resistance confirmed versus Timaru where it was 20 of 35 farms. There was however no significant difference (χ-squared = 1.2975, df = 1, p-value = 0.2547).

Industry supplied samples of *Lolium* spp. included cases of resistance to glyphosate (1 farm), ACCase-inhibitors (7 farms) and ALS-inhibitors (8 farms); those that occur in the survey areas have been included in the maps (Figs. 1 & 2). From other parts of the country, we detected nicosulfuron-resistant *Digitaria sanguinalis* from a farm in the Waikato region of the North Island (had 49-87% survival; Appendix S1). We also had *Stellaria media* samples sourced from ryegrass rotation (near Matamata in the North Island) and samples from Ashburton in the South Island (from barley and ryegrass crops) showed resistance to flumetsulam (100% survival), and chlorsulfuron (95% survival).

Table 2. The number of herbicide resistant grass weeds sourced from 87 randomly surveyed wheat and barley farms near Lincoln (52 farms) and Timaru (35 farms) in the South Island of New Zealand. Industry provided samples of suspected resistant plants are also reported but we do not include their region. We report pyroxsulam resistance levels for oats and bromes treated at 30 g/ai ha. Not all farms with surviving weeds sampled could be tested because of germination problems.

Genus	Site of action	Source	Resistant Farms	Tested Farms	Farms with weed	% Resistant of tested	% Resistant

							of surveyed farms
Avena	ACCase	Lincoln	5	23	29	22	10
Avena	ACCase	Timaru	3	14	23	21	9
Avena	ALS	Lincoln	0	5	29	0	0
Avena	ALS	Timaru	1	9	23	11	3
Avena	EPSPS	Lincoln	0	8	29	0	0
Avena	EPSPS	Timaru	0	8	23	0	0
Bromus	ACCase	Lincoln	0	21	21	0	0
Bromus	ACCase	Timaru	0	19	24	0	0
Bromus	ALS	Lincoln	0	18	21	0	0
Bromus	ALS	Timaru	2	20	24	10	6
Bromus	EPSPS	Lincoln	0	21	21	0	0
Bromus	EPSPS	Timaru	0	14	24	0	0
Lolium	ACCase	Lincoln	7	28	30	25	13
Lolium	ACCase	Timaru	8	18	26	44	23
Lolium	ALS	Lincoln	12	28	30	43	23
Lolium	ALS	Timaru	12	18	26	67	34
Lolium	EPSPS	Lincoln	0	27	30	0	0
Lolium	EPSPS	Timaru	0	18	26	0	0
Phalaris	ACCase	Lincoln	1	10	10	10	2
Phalaris	ACCase	Timaru	0	5	7	0	0
Phalaris	ALS	Lincoln	2	9	10	22	4
Phalaris	ALS	Timaru	3	6	7	50	9
Phalaris	EPSPS	Lincoln	0	6	10	0	0
Phalaris	EPSPS	Timaru	0	4	7	0	0
Vulpia	ACCase	Lincoln	0	3	6	0	0
Vulpia	ACCase	Timaru	0	8	8	0	0
Vulpia	EPSPS	Lincoln	0	6	6	0	0
Vulpia	EPSPS	Timaru	0	7	8	0	0
Vulpia	PSII	Lincoln	0	3	6	0	0
Vulpia	PSII	Timaru	0	6	8	0	0
Avena	ACCase	Industry	1	3	3	33	NA
Avena	ALS	Industry	0	2	2	0	NA
Avena	EPSPS	Industry	0	1	1	0	NA
Bromus	ALS	Industry	0	1	1	0	NA
Digitaria	ALS	Industry	3	3	3	100	NA
Lolium	ACCase	Industry	7	11	11	64	NA
Lolium	ALS	Industry	8	11	11	73	NA
Lolium	EPSPS	Industry	1	9	9	11	NA
Phalaris	ACCase	Industry	0	1	1	0	NA
Phalaris	ALS	Industry	1	1	1	100	NA
Phalaris	EPSPS	Industry	0	1	1	0	NA

Discussion

This study is the first random survey carried out in New Zealand to detect herbicide resistance for a range of arable weeds and estimate its prevalence on wheat and barley farms. Such surveys may not have been implemented previously because costs of these investigations are prohibitive, an earlier estimate suggested it could cost as much as 759 NZD (New Zealand Dollars) per farm [23]. However, we estimated costs of approximately 370 NZD per farm in the second year of our surveys. Despite achieving a sampling of >20% wheat and barley farms in the targeted regions, our estimated prevalence of 48% is likely to be lower than the true rate since detection is imperfect [23]. The basis for this argument is that we could have missed individual resistant plants in a field because we focused on just two fields per farm, and we only sampled weeds that were visible to collectors prior to harvest. Species previously identified as having an elevated risk of developing herbicide resistance in wheat and barley fields were confirmed resistant, i.e., *Lolium multiflorum, Lolium perenne, Avena fatua, Phalaris minor,* and *Stellaria media* [6]. *Bromus catharticus* was resistant to ALS-inhibiting herbicides in this study, a first globally, but identified as medium to low risk by Ngow et al [6].

Before our survey was started, we believed there was a low prevalence of herbicide resistance in wheat and barley, for example, in our funding proposal for this work we estimated that 5-10% of farms would contain resistant weeds. Only three previous publications documented cases of *Lolium* spp. or *A. fatua* resistant to ALS and ACCase herbicides in wheat and barley crops in New Zealand [11,12,24]. There was also a case of resistance of *Stellaria media* in an oat crop [13]. However, we found that resistance is common overall (48%), and particularly for grass weeds on wheat and barley farms with plants surviving on between 23% and 36% of the farms after treatments with ACCase- and ALS-inhibiting herbicides, respectively. This suggests that this issue was historically under-reported by farmers, agricultural chemical suppliers, and consultants as well as under-investigated by scientists.

Because there was variation in effectiveness within herbicides that share the ACCase-inhibitors, this suggests different mutations could be involved [25]. For the ALS-inhibiting herbicides effective on grasses we did not try many of subtypes, e.g. triazolopyrimidine (pyroxsulam), and sulfonylurea (iodosulfuron), so rates and types of cross-resistance are less clear. For the ACCase herbicides, some *A. fatua* oats survived fenoxaprop, but other herbicides within the same mode-of-action (clethodim, haloxyfop, and pinoxaden) remained

effective where they were tested. Populations of *A. fatua* in Australia with resistance to fenoxaprop but not to other with ACCase-inhibiting-inhibiting herbicides, had a mutation at the Trp-1999-Cys site of the acetyl-CoA carboxylase coding region [26]. All *Lolium* spp. collected in the random survey died when treated with glyphosate. Clethodim was usually effective, but three farms had a population resistant to clethodim, pinoxaden and haloxyfop, perhaps implying an Ile-1781-Leu mutation or non-target site resistance [25]. We also documented the first New Zealand cases of *Phalaris minor* surviving treatments with ACCase-inhibitors (fenoxaprop and clodinafop) and ALS- inhibitors (pyroxsulam) , and of *Bromus catharticus* surviving pyroxsulam. Other brome species in the United Kingdom are known to have developed both target and non-target site resistance to ALS-inhibitors [27]. In the case of *Phalaris minor*, the herbicides haloxyfop, clethodim and glyphosate were effective for control of the populations resistant to pyroxsulam, fenoxaprop or clodinafop. Australia is also seeing *Sonchus* spp. with resistance to chlorsulfuron [28]. Rates of resistance in *Lolium* spp. are lower here than in *Lolium rigidum* populations from mainland Australia [29] but similar to rates seen in Tasmania [30].

Industry agronomists supplied us with ryegrass samples suspected of resistance in the field and we confirmed resistance to ALS-inhibitors, ACCase-inhibitors and glyphosate (Table 1). The latter case represented the first case of glyphosate resistant ryegrass plants sourced from a cereal crop (barley) in New Zealand, previous cases had been sourced from vineyards [31]. Other industry supplied samples of *Stellaria media* led to us confirming resistance to flumetsulam and chlorsulfuron sourced from one farm in the Waikato and one farm in the Canterbury regions of New Zealand (in ryegrass and barley fields respectively). Similar resistance was reported from wheat fields in Canada [32]. Chlorsulfuron-resistant *S. media* from an oat field had been documented previously in Southland, New Zealand [13].

The herbicide rates we applied were the highest manufacturer recommended label rates for the herbicides registered in New Zealand. The label rates in New Zealand are often higher than those for other countries. For example, the highest recommended rates for controlling *Lolium multiflorum* in New Zealand is 1458 g ai/ha for glyphosate, 240 g ai/ha for clethodim, and 125 g ai/ha for haloxyfop, while the highest recommended rates on similar Australian product labels are 540 g ai/ha, 60 g ai/ha, and 54 g ai/ha, respectively. The discriminating doses used in other studies with similar systems therefore could be quite different [29,30]. Because of high *Avena fatua* survival rates for the pyroxsulam at the recommended rate of 15 g ai/ha, we ended up respraying all our *A. fatua* populations at two times the label rate (30 g ai/ha), with the non-ionic adjuvant at 0.25% (linear alcohol

ethoxylate 935 g/L); only one population was resistant at that rate. The Rexade GoDri® label in New Zealand suggests 15 g ai/ha rate is effective, but specifically in the presence of crop competition.

We learned a few lessons in the process of doing this work. In the first year of the survey, we bulked samples for multiple plants of the same species in a field, as described for survey work done in Australia [29]. The bulking of samples from a field was found to be a poor sampling strategy compared to sampling seeds from individual mother plants. We found that the cases of resistance were more obvious with samples from individual mother plants – for example, samples usually had >75% survival if they survived a treatment, which is expected because of the shared parentage of the seeds. If we mixed samples from multiple parents, the amount of resistant detected could vary and would depend on the proportion of plants in the field that were resistant. We do not present it here, but it is also possible to see how many of the collected mother plants produce resistant progeny for any given farm. At Ruakura, in the second year of testing, we moved from planting out samples into individual pots to planting into a single propagation tray with six lanes of seedlings planted out, where each lane was a seed from a different sample and every tray could then have a susceptible population included.

Future work will include inheritance studies [33], genetic tests of target site [34,35] and an examination of non-target site [36] genes using weeds surviving treatments in this study and dose response tests for some cases. We will also conduct tests of tillered plants to see if individuals in the resistant populations display resistance to multiple modes-of-action, as well as random surveys in other crop types, e.g., vineyards and maize.

Acknowledgements

First and foremost, we thank the farmers who gave us access to their farms; FAR (Foundation for Arable Research) staff Chelsea Dines, Alex Prince and Harry Washington for collecting the samples.

References

1. Oerke E-C. Crop losses to pests. The Journal of Agricultural Science. 2006;144: 31–43. doi:10.1017/S0021859605005708

2. Stephenson GR. Pesticide Use and World Food Production: Risks and Benefits. Expert Committee on Weeds - Comité d'experts en malherbologie. Banff, Canada: Canadian Weed Science Society; 2000. pp. 9–15. doi:10.1021/bk-2003-0853.ch015

3. Thorne ME, Young FL, Pan WL, Alldredge JR. No-till spring cereal cropping systems reduce wind erosion susceptibility in the wheat/fallow region of the Pacific Northwest. Journal of Soil and Water Conservation. 2003; 9.

4. Baucom RS. The remarkable repeated evolution of herbicide resistance. American Journal of Botany. 2016;103: 181–183.

5. Heap I. International survey of herbicide resistant weeds - weedscience.org. 2021 [cited 25 Oct 2020]. Available: http://www.weedscience.org/

6. Ngow Z, Chynoweth RJ, Gunnarsson M, Rolston P, Buddenhagen CE. A herbicide resistance risk assessment for weeds in wheat and barley crops in New Zealand. Uludag A, editor. PLoS ONE. 2020;15: e0234771. doi:10.1371/journal.pone.0234771

7. Millner JP, Roskruge NR. The New Zealand arable industry. In: Dymond JR, editor. Ecosystem services in New Zealand – conditions and trends. Manaaki Whenua Press, Landcare Research; 2013. pp. 102–114.

8. Biogro. Biogro Certification. In: Biogro Certification [Internet]. 2020 [cited 18 Nov 2020]. Available: https://www.biogro.co.nz/find-certified-organic-licensees-and-products

9. Darling W. The highest barley yield. In: Guinness World Records [Internet]. 23 Jan 2015 [cited 13 May 2020]. Available: https://www.guinnessworldrecords.com/world-records/68919-highest-barley-yield

10. Watson E. The highest wheat yield. In: Guinness World Records [Internet]. 17 Feb 2017 [cited 13 May 2020]. Available: https://www.guinnessworldrecords.com/world-records/highest-wheat-yield

11. Gunnarsson M, James TK, Chynoweth RJ, Rolston MP. An evaluation of the resistance of annual and perennial ryegrass to herbicides. New Zealand Plant Protection. 2017;70: 165–170.

12. Harrington KC, Chynoweth RJ. Have wild oats in Canterbury become resistant to herbicides? New Zealand Plant Protection. 2014;67: 331.

13. Seefeldt S, Peters E, Armstrong ML, Rahman A. Cross-resistance in chlorsulfuron-resistant chickweed (*Stellaria media*). New Zealand Plant Protection. 2001;54: 157–161. Available: http://www.nzpps.org/nzpp_abstract.php?paper=541570

14. Ghanizadeh H, Harrington KC. Herbicide resistant weeds in New Zealand: state of knowledge. New Zealand Journal of Agricultural Research. 2019; 1–12. doi:10.1080/00288233.2019.1705863

15. Hampton JG, Rolston MP, Pyke NB, Green W, Lincoln A. Ensuring the long term viability of the New Zealand seed industry. Agronomy New Zealand. 2012; 12.

16. Busi R, Powles SB, Beckie HJ, Renton M. Rotations and mixtures of soil- applied herbicides delay resistance. Pest Manag Sci. 2019; ps.5534. doi:10.1002/ps.5534

17. Panozzo S, Scarabel L, Collavo A, Sattin M. Protocols for Robust Herbicide Resistance Testing in Different Weed Species. JoVE (Journal of Visualized Experiments). 2015; e52923. doi:10.3791/52923

18. Ghanizadeh H, Harrington KC, James TK, Woolley DJ. Confirmation of glyphosate resistance in two species of ryegrass from New Zealand vineyards. New Zealand Plant Protection. 2013;66: 89–93.

19. Ghanizadeh H, Harrington KC. Fitness costs associated with multiple resistance to dicamba and atrazine in Chenopodium album. Planta. 2018 [cited 6 Dec 2018]. doi:10.1007/s00425-018-3040-5

20. Kahle D, Wickham H. ggmap: Spatial Visualization with ggplot2. The R Journal. 2013;5: 144–161. Available: https://journal.r-project.org/archive/2013-1/kahle-wickham.pdf

21. Wickham H, Averick M, Bryan J, Chang W, McGowan LD, François R, et al. Welcome to the tidyverse. Journal of Open Source Software. 2019;4: 1686 (1–6). doi:10.21105/joss.01686

22. R Core Team. R: A Language and Environment for Statistical Computing. Vienna, Austria: R Foundation for Statistical Computing; 2020. Available: https://www.R-project.org/

23. Buddenhagen CE, Gunnarsson M, Rolston P, Chynoweth RJ, Bourdôt G, James TK. Costs and risks associated with surveying the extent of herbicide resistance in New Zealand. New Zealand Journal of Agricultural Research. 2019;0: 1–19. doi:10.1080/00288233.2019.1636829

24. Ghanizadeh H, Harrington KC, Mesarich CH. The target site mutation Ile-2041-Asn is associated with resistance to ACCase-inhibiting herbicides in *Lolium multiflorum*. New Zealand Journal of Agricultural Research. 2019;63: 416–429. doi:10.1080/00288233.2019.1620296

25. Beckie HJ, Tardif FJ. Herbicide cross resistance in weeds. Crop Protection. 2012;35: 15–28. doi:10.1016/j.cropro.2011.12.018

26. Liu W, Harrison DK, Chalupska D, Gornicki P, O'Donnell CC, Adkins SW, et al. Single-site mutations in the carboxyltransferase domain of plastid acetyl-CoA carboxylase confer resistance to grass-specific herbicides. Proceedings of the National Academy of Sciences. 2007;104: 3627–3632. doi:10.1073/pnas.0611572104

27. Davies LR, Onkokesung N, Brazier- Hicks M, Edwards R, Moss S. Detection and characterization of resistance to acetolactate synthase inhibiting herbicides in *Anisantha* and *Bromus* species in the United Kingdom. Pest Manag Sci. 2020;76: 2473–2482. doi:10.1002/ps.5788

28. Merriam AB, Boutsalis P, Malone J, Gill G, Preston C. Extent of herbicide resistant common sowthistle (*Sonchus oleraceus*) in southern Australia. 21st Australasian Weeds Conference. Sydney; 2018. pp. 16–19.

29. Boutsalis P, Gill GS, Preston C. Incidence of Herbicide Resistance in Rigid Ryegrass (*Lolium rigidum*) across Southeastern Australia. Weed technol. 2012;26: 391–398. doi:10.1614/WT-D-11-00150.1

30. Broster JC, Koetz EA, Wu H. Herbicide resistance frequencies in ryegrass (*Lolium* spp.) and other grass species in Tasmania. Plant Protection Quarterly. 2012;27: 36–42.

31. Ghanizadeh H, Harrington KC, James TK. Glyphosate-resistant Italian ryegrass and perennial ryegrass in New Zealand – a review. New Zealand Plant Protection. 2016;69: 246–251.

32. Laforest M, Soufiane B. Coevolution of Two Sulfonylurea-Resistant Common Chickweed (*Stellaria media*) Biotypes with Different Mutations in the Acetolactate Synthase Gene. Weed Sci. 2018;66: 439–445. doi:10.1017/wsc.2018.26

33. Ghanizadeh H, Buddenhagen CE, Harrington KC, James TK. The Genetic Inheritance of Herbicide Resistance in Weeds. Critical Reviews in Plant Sciences. 2019;38: 295–312. doi:10.1080/07352689.2019.1665769

34. Loureiro I, Rodríguez-García E, Escorial C, García-Baudín JM, González-Andújar JL, Chueca MC. Distribution and frequency of resistance to four herbicide modes of action in Lolium rigidum Gaud. accessions randomly collected in winter cereal fields in Spain. Crop Protection. 2010;29: 1248–1256. doi:10.1016/j.cropro.2010.07.005

35. Malone JM, Boutsalis P, Baker J, Preston C. Distribution of herbicide-resistant acetyl-coenzyme A carboxylase alleles in Lolium rigidum across grain cropping areas of South Australia. Weed Research. 2013;54: 78–86. doi:10.1111/wre.12050

36. Ghanizadeh H, Harrington KC. Non-target Site Mechanisms of Resistance to Herbicides. Critical Reviews in Plant Sciences. 2017;36: 24–34. doi:10.1080/07352689.2017.1316134

Supporting Information

Additional supporting information may be found in the online version of this article:

Appendix S1 Treatment (active ingredient) and survival by farm code and sample number, representing a detailed breakdown of the results presented in Table 2.

Figure 1

Click here to access/download;Figure;2021-07-15 A resistant weed is present pace.tif

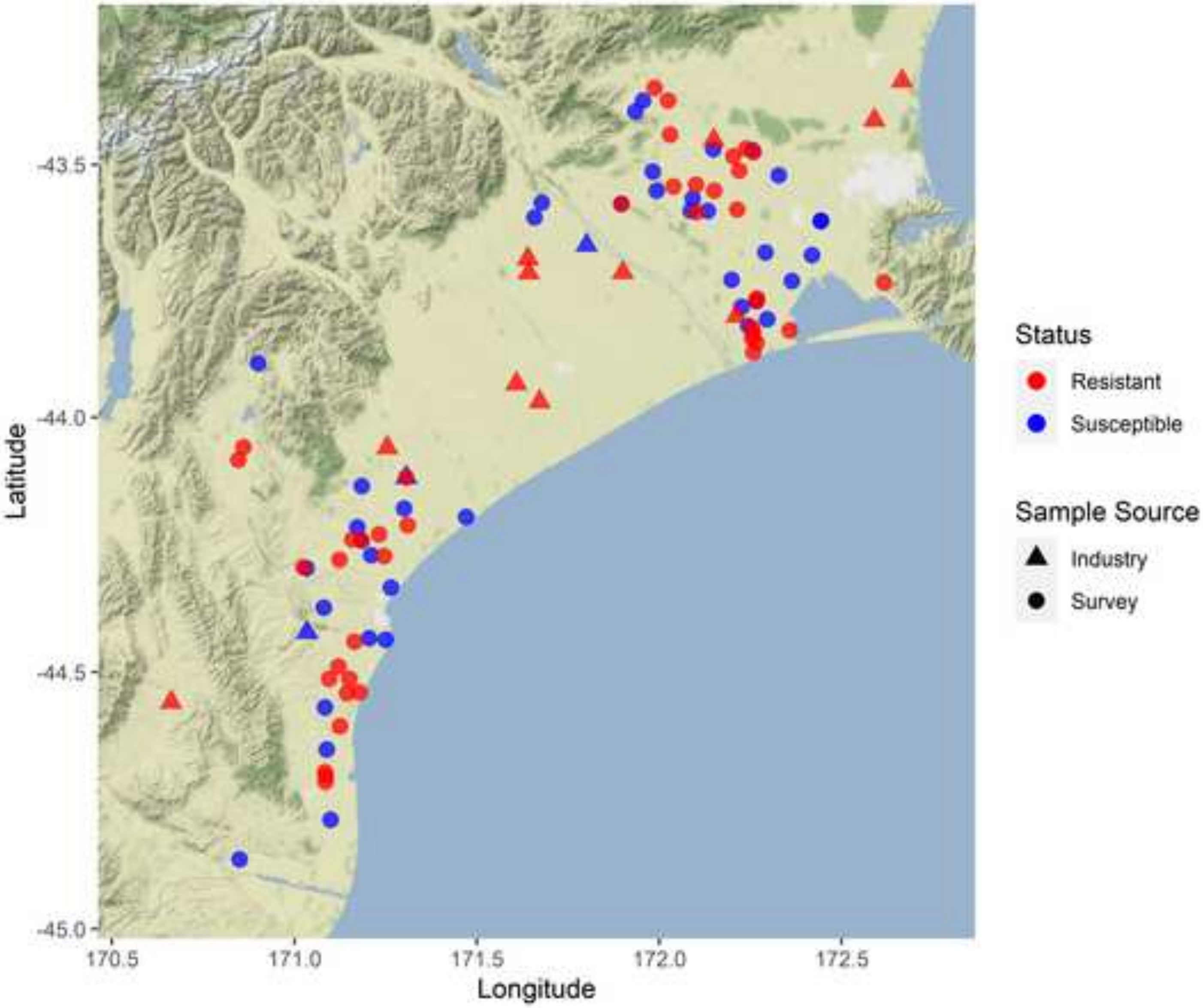

Figure 2

Click here to access/download;Figure;2021-07-15 weed genus resistant grasses_pace.tif

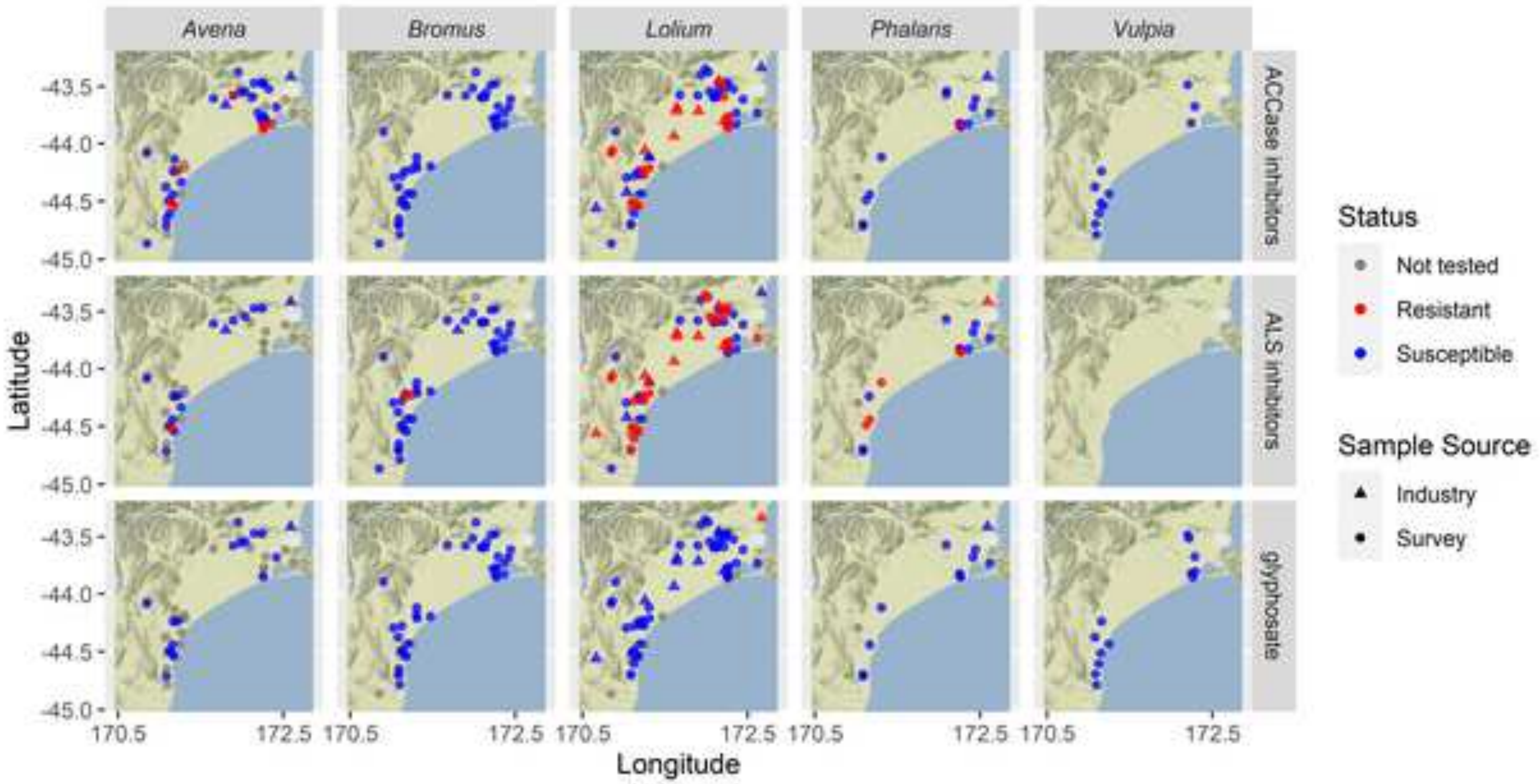

Click here to access/download
Supporting Information
Appendix_S1.csv
