# Supplementary material for: Resistance to post-emergent herbicides is becoming common for grass weeds on New Zealand wheat and barley farms
Source: PLoS One. 2021 Oct 14;16(10):e0258685. doi: 10.1371/journal.pone.0258685 (PMC8516262; doi:10.1371/journal.pone.0258685)
Supplement: Attachment — Submitted filename: Response to Reviewers.docx [file pone.0258685.s003.docx]

**Response to Reviewers**

**Reviewer comments**

Additional Editor Comments: I think this manuscript is a good one although a reviewer rejected. Please, use indirect sentences as a referee pointed out. You maybe need to explain why you had only one sample and tell the readers it cannot create problem for assessing resistance distribution. I am not sure but maybe you can add confidence levels depending on the plants that you collected seeds. ! plant from a field can be considered confidence level 1, and with maximum level confidence level 4 and two more levels or you can make it less or more levels.

*Response:*

*Indirect sentences implemented.*

*Some sentences added about sample sizes (first paragraph of Materials and Methods).*

*The supplemental data can be viewed by readers to see how many plants survived any treatment from any given farm. We chose a single threshold as a conservative measure of prevalence. We’ve added explanations in the text about our focus and added an uncertainty 95% confidence interval for the binomial estimate.:*

*Lines 126-165: ”* *We focused on detecting the presence of resistant plants of any weed detected at the level of farms, not on within farm population level differences. Our sampling rates are more suited to the reliable detection of outcrossing species e.g. Lolium [18,19] , but the presence of each weed species within farms varied stochastically, and time and resource considerations came into play. Combined with our focus on just two fields, we accepted that our estimates of resistance prevalence in farms would be conservative (lower than the true rate).”*

*lines 529-544 “This work was focused on a binary question of whether there is resistance at the level of farms not prevalence within farms…”*

Reviewer #1: Manuscript PONE-D-21-22929 is an elegant report of field surveys of weed populations resistant to ALS, ACCase and EPSPS inhibitor herbicides in wheat and barley fields in New Zealand. Buddenhage et al., in addition to confirming the herbicide resistance of different grassweeds, they also present the distribution maps of the main genus throughout New Zealand. The manuscript is well written, and although the work had its limitations, the authors have reiterated them throughout the text. The authors sampled 20% of the wheat and barley farms, which seems quite representative; and although the authors mention that the high incidence of resistance they found (48%) could be well above the real frequency, since they considered only visible plants before harvest (L318). However, what caught my attention is the number of plants collected per species collected to obtain seeds (1-10 plants, L105). In particular, I considered that this small sample size is not representative for each field, because according to Burgos (2015, 10.1614/WS-D-14-00019.1), although it is possible to work with seeds of 5-10 plants to cross-pollinating species, it is advisable to collect between 20-40 plants or 5000 seeds for self-pollinating species.

Otherwise, the manuscript has many typographical errors that are easily corrected with a careful review. Most of the small typographical errors are marked in the attached manuscript, but personally I suggest that the authors change all expressions made in second person (we, our) to third person.

*Response: The reviewer points out that we are aware of imperfect detection and the true rate of resistance could be higher than we estimated we’ve added the Burgos and mentioned the outcrossing issue. Our ability to find enough weeds for any given species was dependent on its abundance so this is not entirely in our control either.*

Reviewer #2: Methodology inappropriate. Methodology should include genetic confirmation of resistance. Number of testing to low to confirm resistance. Poor results. Inappropriate writing style. Citations occasionally wrong.

*Response: Genetic confirmation of resistance is a useful line of evidence. As the editor will know the methods work well for some target site mechanisms, but are not reliable for non-target site mechanisms. Our methods are classical bioassays widely used and should be acceptable as a means of identify cases with resistance for example see the Burgos and Panozzo article we cite.*

*I did not see any mention of specific problems with the citations but have gone through those to check them. See changes to the citations under a separate heading below.*

**PDF edits**

The edits are visible in the track changes version of the document, where specified line numbers refer to the lines on track changes document.

Indirect sentences added wherever “We” was highlighted.

Where appropriate we changed the Latin names to use an abbreviation for the genus (at second mention). We also added full Latin nomenclature for crops mentioned.

Line 14: Corresponding authors now includes email only.

Line 31-32: herbicide active ingredients are listed (this is all covered in Table 1, and is species specific while the abstract is framed generally).

Line 37: You asked that this be added to the abstract from the results section.

Lines 73-87 first paragraph of introduction: several typos e.g. species names were corrected.

Line 92: Reference **15 Hampton** moved to end of sentence.

Lines 115-121: Indirect style implemented

Lines 125-165: Added sentences about the choices we made regarding the number of plants sampled. This included the addition of two Burgos references.

Lines 167-248: Typos, genus abbreviations and units litres to L as recommended.

Line 267 caption Table 1: bolded the title, reworded, added the adjuvant ingredients reference.

Table 1: adjuvant ingredients added at first mention in the table.

Line 253-260: indirect style

Lines 293-305: Statistical analysis. Copyright information for OpenStreetMaps added for map figures per instructions at <https://journals.plos.org/plosone/s/figures> and the OpenStreetMap website. Also binomial.test for confidence interval estimation.

Lines 307: First paragraph of results. Third person style implemented, crop taxa and genus abbreviations

Line 419: Table 2 added explanation for Site of action abbreviations.

Figure Captions: bolded first sentence, reworded and copyright wording added per recommendation see above.

Lines 363-409: The binomial 95% confidence interval was added for prevalence.

Lines 435: Discussion Reworded the sentence about our prevalence estimate and added the Burgos references [18-19]. Used genus abbreviations.

Lines 529-544: Clarified the limitations of our conclusions in response to reviewer concerns about sample size, while also outlining our change to sampling seed from individual mother plants instead of bulking samples.

Lines 545-552: Indirect style. Addition of a citation to work that raises the possibility that some resistant weeds are arriving in New Zealand from seed contaminants – a new line of investigation that we are pursuing.

**Reference changes**

[2] Stephenson – change case of title

[3] Thorne et al. – added missing page numbers and issue

[5] Heap – changed date accessed

[14] Ghanizadeh & Harrington – add issue, year and page numbers (I was citing early online version).

[15] Hampton et al – added missing page numbers and issue

[16] Busi et al – added page numbers and issue

[17] Holden – added new reference to NZ agrichemical manual

[18] Burgos – new reference

[19] Burgos et al – new reference

[20] Panozzo et al – added issue/volume change case

[22] Ghanizadeh & Harrington – add pages volume date

[23] Kahle & Wickam – change case

[27] Buddenhagen et al – page numbers date and volume

[33] Boutsalis et al – change case

[36] Laforest et al – change case of title

[37] Ghanizadeh et al – change case of title

[39] Malone et al – italics for species.

[40] Ghanizadeh & Harrington – change case of title.

[41] Rubenstein et al – new reference.
