# Supplementary material for: Resistance to post-emergent herbicides is becoming common for grass weeds on New Zealand wheat and barley farms
Source: PLoS One. 2021 Oct 14;16(10):e0258685. doi: 10.1371/journal.pone.0258685 (PMC8516262; doi:10.1371/journal.pone.0258685)
Supplement: Attachment — Submitted filename: Response to Reviewers.docx [file pone.0258685.s005.docx]

21 September 2021

Dear PLOS,

Please consider our revised research article “**Resistance to post-emergent herbicides is becoming common for grass weeds on New Zealand wheat and barley farms**.” We thank the editor and reviewers for the helpful comments which guided us to make improvements to the manuscript.

We had a prior interaction with PLOS about this manuscript – the original manuscript was submitted with cover letter 14 April 2021, and we had a reply requesting revisions in an email from the editor on 20 August 2021, followed by a decision: revision required PONE-D-21-22929R1 on 18 September 2021.

Most edits made using track changes by the reviewers accepted. Other suggestions were implemented unless indicated otherwise. Itemized responses to the reviewers comments follow in this letter.

Sincerely,

Chris Buddenhagen PhD

chris.buddenhagen (skype)

+64 221 04084 (cell)

[Chris.Buddenhagen@agresearch.co.nz](mailto:Chris.Buddenhagen@agresearch.co.nz)

**Response to Reviewers**

**Reviewer comments**

Editor Comments: Could you please give attention to typos. I suggest please avoid wording such as resistant farms. - *Done*

*Response:*

Reviewer #1: The authors have satisfactorily addressed most of the comments in the previous version and intellectually the manuscript is acceptable for publication; however, I still found various typos throughout the manuscript.

*L28:* Glyphosate’s mode of action can be added. *– Although there is only one herbicide with this mode of action, we added it as requested by a reviewer. It seems unnecessary.*

L33: Resistant farms???? *– reworded to indicate the weeds are resistant*
Maybe: Weeds, such as Avena fatua (9%, 1%, 0% of farms), Bromus catharticus (0%, 2%, 0%), Lolium spp. (17%, 28%, 0%), Phalaris minor (1%, 6%, 0%), and Vulpia romoides (0%, not tested, 0%), from different farms (denominator is 87 farms) displayed resistance to ACCase-inhibitors, ALS-inhibitors, and glyphosate *– reworded to indicate the weeds are resistant*

L38: change Phalaris to P. *– changed*
L39: Bromus to B. *– changed*
L41: Sonchus asper but also S. oleraceus. *– changed*
L41: Check double point *– changed*
L43: Phalaris to P.v*– changed*
L84: [17]space(compared *– changed*

*L84: Reviewer comment:* Since the sentence, starting with “Another plausible expalanation…” gives a reason for low number of resistance. It is understood that higher application rates is one of reason for low resistance cases. It can not be the reason for low number of resistance. I couldn’t see any discussion about this sentence at Discussion part below, either.

*Response:*

*Herbicides applied at registered rates can clearly select for major gene (e.g., target-site) resistance, whereas initially, suboptimal herbicide rates may select for both major and minor gene (i.e. quantitative) resistance. We discuss the difference in rates between Australia and NZ later. We would prefer to leave this sentence in the article.*

L116: *Lolium multiflorum* (when the scientific name begins the phrase, the genus is spelled out in full) *– changed*
L138: Canterbury). *– changed*
L156, L193 and L286: Avena fatua *– changed*

L163: Reviewer comment: These sentences can be converted into a table with information on the name of these weed species, sample size, where it was collected, which herbicide or mode of action it was tested against

*Response:*

*We would like to leave this here- it draws a distinction between the samples processed in the random survey and the ones that were supplied as suspected cases of resistance by industry agronomists, and it includes well as one broadleaf weed that we tested. The grasses are also in Table 2 and all are in the Appendix S1.*

*159 – moved the sentences about susceptible controls to the “Collection of plant material” section per recommendation.*
L181: Sonchus asper and S. oleraceus (if the mention of species has no hierarchy, they are listed alphabetically) *– changed [though this is not a rule I have heard before]*
L211-213: abbreviate the genus of the species *– changed*
L266 and L341: list the scientific names alphabetically *– changed*
L341: Bromus …*– changed*

L269: *Reviewer comment*: In order to be able to talk about herbicide resistance, discriminating doses and label doses should be used for each active substance and each population in pot studies. I have seen that you used more than one dose for some weeds. But to make confirmation of herbicide resistance for all the weeds you studied, more comprehensive studies must be performed. So instead of using certain statement of herbicide resistance, suspicion of herbicide resistance should be mentioned for weeds and active ingredients. This study is a kind of preliminary study of finding suspicious herbicide resistance biotypes.

Also in the email from the editor this comment is similar:

*Reviewer #3 comment*: This study is an important and intensive survey for resistance weed species but it is a kind of preliminary study of finding suspicious herbicide resistance biotypes. Since the sample size and treatments is not enough, the conclusions should be reconsidered to mention herbicide resistance. Minor revisions were made in the text.

*Response*:

*By the definition the recommended dose is a discriminating dose, it is the dose the manufacturer has determined to kill the weeds on the label. When we detect resistant plants there is every reason to believe they are resistant. We experimentally confirmed the resistance by spraying with this dose and it did not kill the resistant plants indicated here, but it did kill susceptible reference populations. We are not the first to use the label rate as a discriminating dose: Broster et al 2012 cited, and the following references.*

*Broster JC, Koetz EA, Wu H 2010. A survey of southern New South Wales to determine the level of herbicide resistance in brome grass and barley grass populations. In: Zydenbos SM ed. Christchurch, New Zealand. Seventeenth Australasian Weeds Conference: 274–277.*

*Broster JC, Koetz EA, Wu H 2011. Herbicide resistance levels in annual ryegrass (Lolium rigidum Gaud.) in southern New South Wales. Plant Protection Quarterly 26: 22–28.*

*Owen MJ, Martinez NJ, Powles SB 2014. Multiple herbicide-resistant Lolium rigidum (annual ryegrass) now dominates across the Western Australian grain belt. In: Iannetta P ed. Weed Research 54: 314–324.*

*Owen MJ, Martinez NJ, Powles SB 2015a. Herbicide resistance in Bromus and Hordeum spp. in the Western Australian grain belt. Crop and Pasture Science 66: 466.*

*We responded to the idea that the sample size is inadequate in the last peer review by indicating that our estimate of the number of farms with resistant plants is likely conservative. Bear in mind that even with much higher sampling rates, you would still be unable to determine if you detected all cases of resistance that might be present. We do not exaggerate our claims about resistance rates.*

*We state this:*

*In the methods we state this:*

*Our sampling rates are more suited to the reliable detection of outcrossing species e.g., Lolium [18,19], but the presence of each weed species within farms varied stochastically, and time and resource considerations came into play. Combined with our focus on just two fields, we accepted that our estimates of resistance prevalence in farms would be conservative (lower than the true rate).*

*In the discussion:*

*After randomly sampling of >20% wheat and barley farms in the targeted regions, resistance was detected in 48% of farms, this is likely to be lower than the true rate since detection is imperfect [27]. The basis for this argument is that we could have missed individual resistant plants in a field and because we focused on up to ten plants in just two fields per farm, depending on which weeds were available to collectors prior to harvest [18,19].*

*We added this in the discussion this time: Our sampling rate is better suited to the detection of outcrossing weed species but could miss some self-pollinating species [18.19].*

L379: *Reviewer comment*: From the beginning I don’t understand whay you tested glyphosate while you are surveying cereal farms, since glyphosate is not recommended to use in cereals. You can add some expalanatory sentences why you choosed glyphosate. For example; plant rotation or resistance seed dispersal.

*Response:*

*Glyphosate is commonly used for seed bed preparation and glyphosate resistant ryegrass is known from elsewhere in New Zealand. Under the section growing plants and spraying the following sentence was added: “Glyphosate was tested because it is commonly used prior to planting for seed bed preparation.” In addition the detection of glyphosate resistant ryegrass in a barley crop proves that we were right to look at this herbicide.*
